# Clinical risk factors of extracorporeal membrane oxygenation support in older adults

**DOI:** 10.1371/journal.pone.0195445

**Published:** 2018-04-06

**Authors:** Te-Chun Yeh, Hsiao-Huang Chang, Luo-Ping Ger, Ju-O Wang, Senyeong Kao, Shung-Tai Ho

**Affiliations:** 1 Graduate Institute of Life Sciences, National Defense Medical Center, Taipei, Taiwan; 2 Academy of Humanities and Innovation, Taipei City Hospital, Taipei, Taiwan; 3 Division of Cardiovascular Surgery, Department of Surgery, Taipei Veterans General Hospital, Taipei, Taiwan; 4 Department of Medical Education and Research, Kaohsiung Veterans General Hospital, Kaohsiung, Taiwan; 5 School of Public Health, National Defense Medical Center, Taipei, Taiwan; 6 Department of Anesthesiology, Taipei Veterans General Hospital, Taipei, Taiwan; Universita degli Studi di Bologna, ITALY

## Abstract

**Background:**

The ageing population and the expected increase in the number of elderly patients make an evidence-based assessment of Extracorporeal Membrane Oxygenation (ECMO) therapy in old patients progressively more important. Veno-arterial (VA) ECMO results for patient aged <65 years is well known. However, the risk profile and in-hospital prognosis of advanced age patients with ECMO still need more investigation. The aim of this study was to identify risk factors that predicted the outcomes for elderly patients who received VA-ECMO.

**Methods:**

In this retrospective study, medical records for patients with ECMO aged 65 years and over were collected between 2009 and 2012 at a tertiary hospital.

**Results:**

A total of 99 patients (mean age: 76.4±6.4 years) were included. The most common condition requiring VA-ECMO support was cardiogenic shock. Among survivors on VA-ECMO, 28 (28.3%) patients were successfully weaned from support. Thirteen (13.1%) patients were successfully discharged. We found that cardiogenic shock (OR = 3.158, P = 0.013), acute physiology and chronic health evaluation II (APACHE II) score (OR = 1.147, P<0.001), and simplified acute physiology score II (SAPS II) score (OR = 1.054, P = 0.001) were risk factors associated with survival on VA-ECMO. By using the areas under the receiver operating characteristic (AUC) curve, the APACHE II score and SAPS II score displayed acceptable discriminative power (AUC 0.722; 0.715, respectively).

**Conclusion:**

Our findings indicate that the risk of mortality increases with cardiogenic shock, higher APACHE II score, and higher SAPS II score. These risk factors can be utilized as potential predictors to identify the potential candidates for ECMO support.

## Introduction

Extracorporeal membrane oxygenation (ECMO) is used for the management of life-threatening cardiac or pulmonary failure. ECMO uses the cardiopulmonary bypass circuit to provide prolonged respiratory or cardiorespiratory support for patients who fail to respond to conventional intensive care management practices like mechanical ventilation, increased oxygenation and medications [1,2]. Even though ECMO is not a disease treatment, it provides additional time to allow for recovery from existing lung and/or cardiac disease [3–5].

Due to the increased number of elderly people in the population, as well as an increased average life expectancy, an evidence-based assessment of ECMO therapy in this group is becoming more critical. According to Hsu et al.,[6] ECMO is used in Taiwanese patients with severe clinical condition to give added benefit, specifically among elder adults. However, there is no definite recommendation for the risk profile and in-hospital prognosis of older adult patients who receive ECMO. Therefore, the risk factors associated with hospital mortality in this population remain unclear. Making better decisions about how to allocate scarce medical resources requires a better understanding of the factors that influence who is likely to survive if given ECMO versus those who would die whether they receive ECMO or not. Therefore, this study was aimed to identify predictors of mortality in elderly patients successfully weaned from veno-arterial (VA)-ECMO.

## Methods

This retrospective, single centre study was approved by the Institutional Review Board (No. 2013-02-011A) of the Taipei Veterans General Hospital, which is a national tertiary hospital in Taiwan. Data were retrieved from patient records with ECMO patients between 2009 and 2012.

### Measures and definitions

Clinical data were obtained from a retrospective review of each patient’s medical records. Patients ≥65 years who received VA-ECMO support between January 2009 and December 2012 were included. Each patient’s pre-ECMO conditions (before ECMO deployment), duration of ECMO therapy, and survival to hospital discharge were collected and evaluated. The following variables were collected: age, gender, hospital stay, ICU stay, main diagnosis (cardiac failure: cardiogenic shock, acute myocardial infarction (AMI), acute resuscitation during CPR, acute myocarditis, septic shock, and pre-heart transplant recipient), underlying diseases (hypertension, diabetes mellitus (DM), end-stage renal disease (ESRD), cancer, congestive heart failure (CHF), cerebral vascular accident (CVA), and coronary artery disease (CAD)), renal failure, liver failure, duration of ECMO therapy (type and durations) and the outcomes (weaning off ECMO and survival to discharge).

The acute physiology and chronic health evaluation II (APACHE II) score [7], multiple organ dysfunction (MOD) score [8], and simplified acute physiology score II (SAPS II) score [9] were calculated using the worst variables recorded within the 24 hours before ECMO initiation to predict risk of hospital mortality.

The APACHE II, SAPS II score and MODS score are the widely used scoring systems in the intensive care unit (ICU). The APACHE II score is a severity of disease classification system developed from a large sample of ICU patients in the United States [7]. To calculate the APACHE II score (range 0 to 71), twelve common physiological, age and 2 disease-related variables are calculated.

The SAPS II score is a severity score and mortality estimation tool development from a large international sample of patients in Europe and North American [9]. The SAPS II score is made of 17 variables: age, 2 physiology variables, type of admission (scheduled surgical, unscheduled surgical, or medical), and three underlying disease variables (acquired immunodeficiency syndrome, metastatic cancer, and hematologic malignancy). The SAPS II score ranges from 0 to 163 points and provides a method to convert the score to probability of hospital mortality.

The MODS was calculated as described by Marshall et al in 1995 using six organ systems [8]. Multiple organ failure is the leading cause of mortality in patients admitted to the ICU.

### Clinical management

The ECMO support is widely used in the majorities of major hospitals in Taiwan. In our hospital, we applied around 200 cases of ECMO per year. Two main types of the devices are used which include (Sorin SCP revolution 5, LivaNova, London, UK and Maquet Rotaflow RF32, Rastatt, Germany). We routinely use peripheral cannulation for all VA-ECMO cases via femoral artery (percutaneously or cut-down) and femoral vein. Distal perfusion catheter to prevent lower limb ischemia is also routinely applied. If the patient has peripheral arterial disease (PAD), we shift the cannulation site to right side axillary artery. After initiation of the support, intravenous heparin is given to maintain activated partial thromboplastin time (aPTT) to 45–60 sec. the target level of hematocrit is above 30% and the platelet count is above 80,000/ml. Most of the time, we still use low to moderate dose of inotrope or vasopressor to maintain adequate whole body perfusion and blood pressure. The blood flow of ECMO is around 2.5–3.5 L/min. Intra-aortic balloon pump (IABP) is routinely used for all cardiogenic shock patient to provide better opportunity of survival. The setting target of the FiO_2_ of the respirator is below 35% if possible. The peripheral artery line is inserted from right side radial artery to provide more accurate information of the blood saturation of coronary artery and right side hemisphere. The pulse oximetry is placed at right side finger under the same concern to prevent possible hypoxic injury of the myocardium and right side hemisphere (the blood directly from the left ventricle might be hypoxia).

The indications for VA-ECMO were the most common: cardiac arrest, refractory cardiogenic shock with maximum inotropic support, failure to wean from cardiopulmonary bypass after cardiac surgery, and bridge to decision for transplant or VAD. VA-ECMO support was contraindicated in patients with unrecoverable cardiac function, patients who are not candidates for transplantation or durable mechanical support, chronic organ dysfunction (emphysema, cirrhosis, renal failure), prolonged cardiopulmonary resuscitation (CPR) without adequate tissue perfusion, and those with compliance limitations (financial, cognitive, psychiatric, and social limitations).

### Statistical analysis

Categorical data were presented as number and percentage. Pearson’s Chi-square test or Fisher’s exact test were used for categorical variables and to compare the differences between survivors and non-survivors. Continuous data were summarized as the mean±SD. Crude and adjusted odds ratios (ORs) and respective 95% confidence intervals were estimated with univariate and multivariate logistic regression analysis in order to examine the risk of in-hospital mortality, age, indications, underlying diseases, comorbidities, and mortality risk scores in VA-ECMO patients.

The ability to predict mortality was evaluated by the receiver operating characteristic (ROC) curve and the areas under the receiver operating characteristics (AUC) curve. ROC and AUC were calculated to determine the diagnostic accuracy of the APACHE II score, MOD score, and SAPS II score. The best predictive cut-off values for risk scores were calculated based on ROC area under the curve analysis with the Youden index (sensitivity + specificity − 1) [10].

A stratified analysis was used to evaluate in-hospital mortality rate among different patient subgroups. All statistical tests were two-tailed, with the level of significance set at a P value <0.05. Data analysis was performed using SPSS for Windows.

## Results

**Fig 1:**
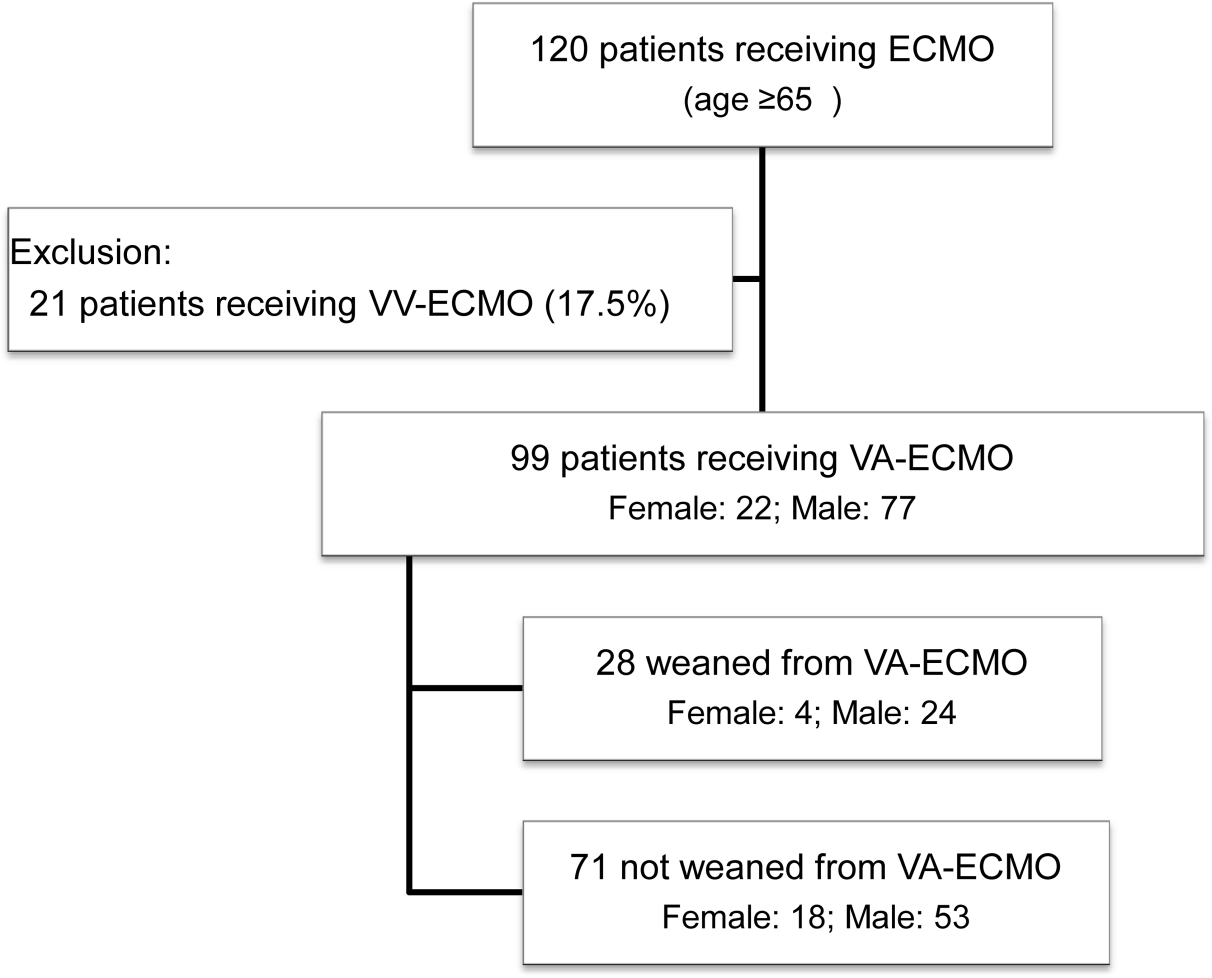
Flow chart of patients received ECMO from 2009–2012.

Among 120 ECMO patients aged ≧65 years during investigation period, 99 (82.5%) required VA-ECMO for hemodynamic instability with or without concomitant respiratory failure. Twenty-one patients (17.5%) who received VV-ECMO for respiratory failure were excluded. A total of 99 patients (22 female, 77 male) received ECMO therapy and were enrolled. Twenty-eight patients (28.3%) were successful weaned from ECMO therapy. Thirteen patients ultimately survived until discharge; the hospital survival rate was 13.1% (Fig 1).

### Patient characteristics

The demographic and clinical characteristics of 99 ECMO patients are described in Table 1. The mean age of patients treated with ECMO was 77.9±6.4 and 75.9±6.4 years for the survival and non-survival groups, respectively. The survival versus the non-survival group had a lower proportion of females (14.3% vs 25.4%), but the differences were not statistically significant (P = 0.355).

**Table 1:** The demographic and clinical characteristics in elderly patients receiving VA-ECMO.

Pre-ECMO Variables	All patients	Successfully weaned from ECMO		P value
	(n = 99)	Survival (n = 28)	non-survival (n = 71)	
Age (years)	76.4±6.4	77.9±6.4	75.9±6.4	0.244
Sex (F/M)	22/77	4/24	18/53	0.355
Hospital stay (Days)	28.7±48.7	69.5±76.2	12.6±12.5	0.001
ICU stay (Days)	17.5±20.6	38.1±24.8	9.4±11.0	<0.001
ECMO support Duration (Days)	5.2±5.2	6.4±4.0	4.7±5.6	0.158
Risk score				
APACHE II score	38.5±7.0	34.3±7.4	40.2±6.1	<0.001
MOD score	11.4±3.3	10.4±3.4	11.9±3.1	0.060
SAPS II score	82.5±15.9	73.6±16.3	86.1±14.4	<0.001

The hospital stay and ICU stay were significantly longer for the survival group than those of the non-survival group (hospital stay: 69.5±76.2 vs 12.6±12.5 days, P = 0.001; ICU stay: 38.1±24.8 vs 9.4±11.0 days, P<0.001). However, there was no significant difference in ECMO support duration between the survival and non-survival groups (6.4±4.0 vs 4.7±5.6 days, P = 0.158). The APACHE II score, MOD score, and SAPS II score were significantly higher for the non-survival group than the scores in the survival group (APACHE II score: 34.3±7.4 vs 40.2±6.1, P<0.001; MOD score: 10.4±3.4 vs 11.9±3.1, P = 0.060; SAPS II score: 73.6±16.3 vs 86.1±14.4, P<0.001).

Table 2 summarizes the results of univariate comparisons of clinical characteristics between patients with hospital survival and hospital non-survival. The most frequent reason (indication) for ECMO support was cardiogenic shock (65.7%), following by acute resuscitation during CPR (49.5%), and AMI (48.5%). The most frequent underlying disease was hypertension (69.7%) followed by renal failure (68.7%) and CAD (54.5%).

**Table 2:** Clinical characteristics of hospital mortality in patients receiving VA-ECMO.

Pre-ECMO Variables	n (%)		Successfully weaned from ECMO	Univariate logistic regression	
				OR	P value
Age	99	(100)		0.960	0.242
Cardiac failure					
Cardiogenic shock	65	(65.7)	13 (20.0)	3.158	0.013
AMI	48	(48.5)	12 (25.0)	1.371	0.482
Acute resuscitation during CPR	49	(49.5)	12 (24.5)	1.451	0.408
Pre-heart transplant recipient	1	(1.0)	0 (0)	-	-
Acute myocarditis	1	(1.0)	0 (0)	-	-
Septic shock	12	(12.1)	3 (25.0)	1.210	0.788
Underlying diseases					
Hypertension	69	(69.7)	18 (26.1)	1.417	0.463
DM	33	(33.3)	10 (30.3)	0.863	0.752
ESRD	17	(17.2)	6 (35.3)	0.672	0.482
Cancer	14	(14.1)	4 (28.6)	0.984	0.979
Heart disease					
CHF	18	(18.2)	5 (27.8)	1.031	0.958
CVA	9	(9.1)	2 (22.2)	1.422	0.673
CAD	54	(54.5)	13 (24.1)	1.577	0.310
Aortic aneurysm	14	(14.1)	4 (28.6)	0.984	0.979
Renal failure (before ECMO)	68	(68.7)	18(26.5)	1.323	0.554
Liver failure (before ECMO)	7	(7.1)	0 (0)		

AMI, acute myocardial infarction; CPR, cardiopulmonary resuscitation; DM, diabetes mellitus; ESRD, end-stage renal disease; CHF, congestive heart failure; CVA, cerebral vascular accident; CAD, coronary artery disease, OR, Odds ratio.

### Predictors of ability to be weaned from VA-ECMO

To determine risk factors associated with in-hospital mortality in ECMO patients, ORs and P values were estimated using univariate logistic regression. These results showed that cardiogenic shock (OR = 3.158, P = 0.013) was significant risk factors on ECMO survival (Table 2).

We further determined the relationship between patients’ risk scores and in-hospital mortality. Table 3 shows that ECMO non-survivors had a significantly higher odds ratio for having a higher APACHE II score, and SPAS II score than those in survivors; the odds ratios of these risk scores were 1.147 and 1.054, respectively.

**Table 3:** Comparison of discrimination of the scoring method in predicting VA-ECMO survival.

	OR	P	ROC curve		Cut-off value	Sensitivity	Specificity
Pre-ECMO Variables			AUC	P			
APACHE II score	1.147	<0.001	0.722	0.001	37.5	0.696	0.714
MOD score	1.159	0.064	0.629	0.070	12.5	0.460	0.760
SAPS II score	1.054	0.001	0.715	0.001	86.5	0.627	0.786

To assess the predictive value of each risk score in predicting in-hospital mortality, the AUC, cut-off value, sensitivity, and specificity are presented in Table 3. When predicting ECMO survival, the APACHE II score AUC was 0.722, and the sum of sensitivity and specificity was maximized at an APACHE II score of 37.5 (sensitivity = 0.696, specificity = 0.714). For SAPS II, the highest value of the Youden index was obtained at a cut-off point of 86.5 (sensitivity = 0.627, specificity = 0.786, AUC = 0.683).

**Fig 2:**
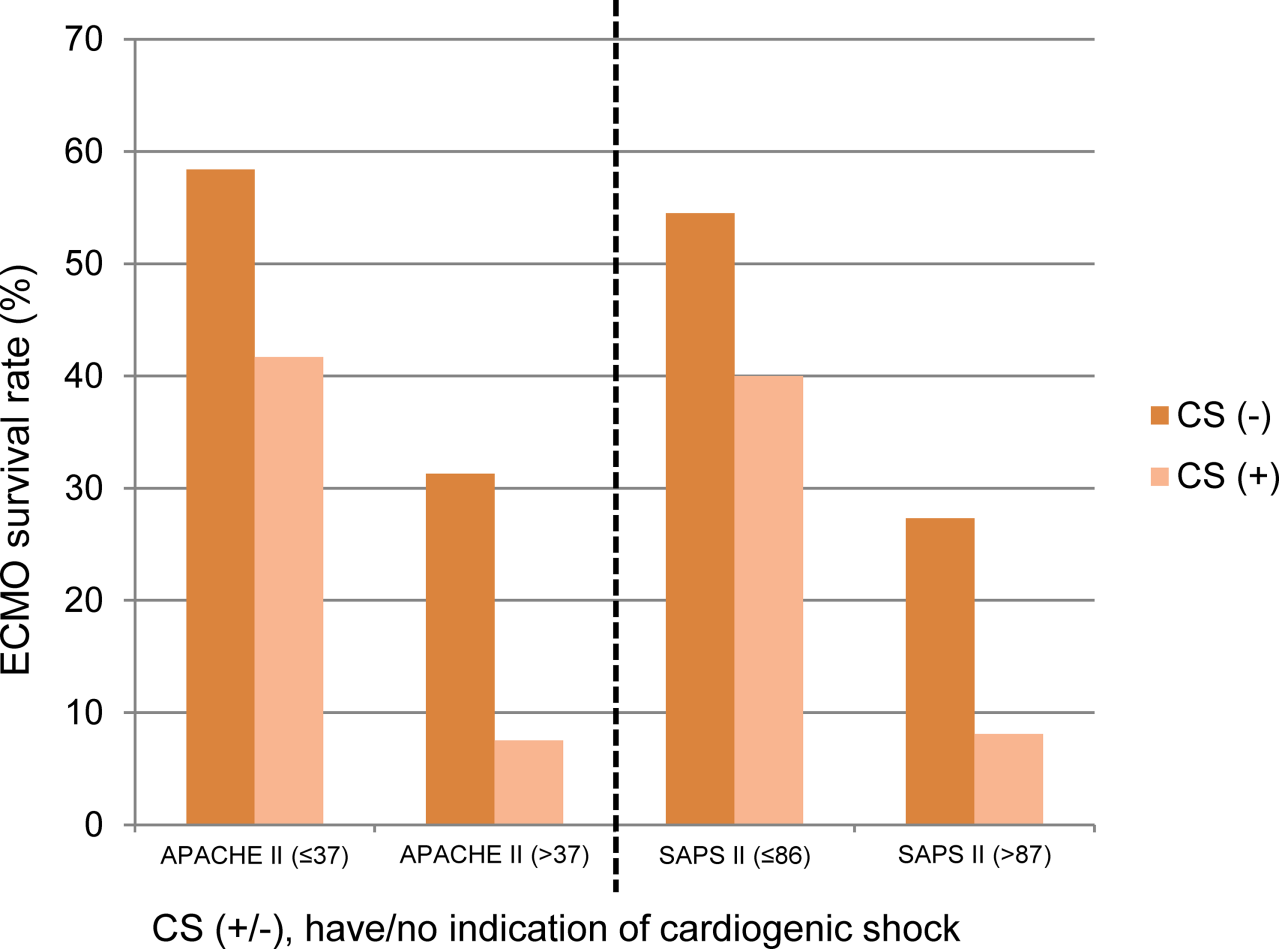
ECMO survival rate in the different patient subgroups with stratification by cardiogenic shock, APACHE II score, and SAPS II score.

Fig 2 depicts ECMO survival rate categorized by dichotomous APACHE II score >37/≤ 37, SAPS II score >86/≤ 86, and have/no indication of cardiogenic shock. Patients with cardiogenic shock and APACHE II score greater than 37 had a significantly poorer ECMO survival rate (7.5%) than patients with no cardiogenic shock and APACHE II score less than or equal to 37 (58.4%). Patients with cardiogenic shock and SAPS II score >86 had a significantly poorer ECMO survival rate (8.1%) than patients with no cardiogenic shock and SAPS II score ≤ 86 (54.5%).

## Discussion

Taiwan is currently facing the challenge of an ageing population and widespread ECMO use in older adults [11]. In this study, our major findings in VA-ECMO patients aged ≧65 years are as follows: (1) cardiogenic shock (OR = 3.158, P = 0.013), APACHE II score (OR = 1.147, P<0.001), and SAPS II score (OR = 1.054, P = 0.001) were risk factors associated with survival on VA-ECMO; (2) the APACHE II score (AUC = 0.722) and SAPS II score (AUC = 0.715) displayed acceptable discriminative power. We believe our study could be helpful in determining which patients should be selected as ECMO candidates before initiation.

With the emergence of value-based healthcare, clinicians need to carefully evaluate older adult patients’ clinical conditions to decide the most appropriate therapeutic approach for ECMO support. Making better decisions when allocating scarce medical resources requires a better understanding of who is likely to survive if given ECMO. Many risk factors may affect clinical outcomes for ECMO patients. Therefore, identifying these factors will provide clinicians with valuable prognostic information when making medical decisions.

In our study, increased risk of not successfully weaned from VA-ECMO was explained by three factors: cardiogenic shock, APACHE II score, and SAPS II score. Although advanced age is a general predictor of hospital mortality in critical ill patients, the effectiveness of ECMO in older adult patients has not been determined. Some previous reports have suggested that ECMO might be less suitable (a contraindication) in older adults [12,13]. Other studies have revealed that ECMO was effective in older adult patients, yielding hospital survival rates similar to the rates for younger patients [14]. Data from most ECMO studies suggest that advanced age is associated with the controversial results in ECMO patients. According to the guidelines published by the Extracorporeal Life Support Organization (ELSO), age is not a specific contraindication for respiratory failure and heart failure but should be considered one because the risk increases with ageing [13,15]. However, the poor prognosis may be due to the higher prevalence of coexisting medical conditions in older adult patients [16]. These results could make clinicians wary of using ECMO. Age itself should not preclude patients from being candidates for ECMO support. Thus, the decision to perform ECMO in older patients should not be based on age alone. For critically ill patients, current research has demonstrated that illness severity, acute physiology, premorbid status, and multiple organ failure have a greater impact on prognosis than age alone [17–19]. These factors could be stronger predictors of hospital mortality than age.

Previous studies [11,12] indicated that cardiogenic shock in patients requiring ECMO support indicated a poor prognosis and were important predictors of hospital mortality. These results are consistent with our study and likely relate to disease processes and other comorbidities.

In the present study, the APACHE II score before ECMO initiation significantly predicted mortality in the critically ill patients (aged≧65 years) who underwent VA-ECMO. The mortality rate increased with an increase in pre-ECMO APACHE II score. A recent prospective study demonstrated that a higher APHCHE II score was associated with unsuccessful weaning from ECMO for ARDS patients [20]. Lin CY et al. showed that an APACHE II score >22 resulted in a higher mortality in critically ill patients (mean age: 47±22 years) undergoing VA-ECMO and VV-ECMO [21]. In a study conducted by Chiu LC et al, the prognostic scores for the APACHE II was 24 for ARDS patients (mean age: 48.0±17.3 years) in the ICU undergoing VV-ECMO [22]. These diverse prognostic values are likely to be influenced by different age groups, severity of diseases, indications for ECMO in each study.

We confirmed that a higher SAPS II score was a good predictor of in-hospital mortality in ECMO patients. A higher SAPS II score before the ECMO procedure, which probably represents a poor overall functional status, was an important risk factor predicting survival. It has been shown that a higher SAPS II score was a predictor of hospital mortality for ECMO patients and has a fair prediction ability [23–26]. The mortality rate increased significantly with an increase in pre-ECMO SAPS II score. In previous literature, a SAPS II score about 80 or above was considered an indicator of favourable outcomes before the use of ECMO in adult patients [23,24]. Therefore, we identified SAPS II as a predictor and suggest a SAPS II value of 86 as the cut off in the aged patient.

In this study, there are several limitations. First, this was a retrospective study performed at a single tertiary-care hospital, limiting the generalization of its findings. Second, different management approaches adopted by physicians may have influenced the study outcome. Third, there are no internal and external validation cohorts for score testing. The score testing for internal validation cohort cannot be performed due to the insufficient sample size. In addition, a multicentered and large scale study with independent cohort is suggested to validate our findings.

## Conclusion

In conclusion, this study observed the in-hospital mortality under VA-ECMO in older adult patients. According to these results, it is still difficult to conclude that ECMO is a recommended therapy for the elderly patients. However, the poor prognosis may be due to the higher prevalence of coexisting medical conditions in older adult patients. ECMO should be performed only in carefully selected patients over 65 years.

Our findings indicate that the risk of mortality increases with cardiogenic shock, high APACHE II score, and SAPS II score and should be taken into account when considering initiation of ECMO support among adults aged 65 and over. This study provides clinicians with valuable prognostic information for medical decision-making.
